# BMI-Dependent Modulation of the Soluble RAGE-Sirtuin-1 Axis by Coffee Type in Coronary Artery Disease

**DOI:** 10.3390/nu18142305

**Published:** 2026-07-14

**Authors:** Alessandra Roggerio, Celia Maria Cassaro Strunz, Gabriela Parisi Polo, Luíz Antônio Machado César, Antonio De Padua Mansur

**Affiliations:** 1 Laboratório de Análises Clínicas, Instituto Do Coracao (InCor), Hospital das Clinicas HCFMUSP, Faculdade de Medicina, Universidade de Sao Paulo, Sao Paulo 05403-900, SP, Brazilcelia.strunz@incor.usp.br (C.M.C.S.); 2 Unidade de Coronariopatia Crônica, Instituto do Coracao (InCor), Hospital das Clinicas HCFMUSP, Faculdade de Medicina, Universidade de Sao Paulo, Sao Paulo 05403-900, SP, Brazil; 3 Serviço de Prevenção, Cardiopatia na Mulher e Reabilitação Cardiovascular, Instituto do Coracao (InCor), Hospital das Clinicas HCFMUSP, Faculdade de Medicina, Universidade de Sao Paulo, Sao Paulo 05403-900, SP, Brazil

**Keywords:** caffeinated coffee, coronary artery disease (CAD), Sirtuin-1, soluble RAGE, obesity

## Abstract

Background: Coffee contains bioactive compounds associated with cardiometabolic benefits, which modulate metabolic pathways of Sirtuin-1. The AGEs–RAGE axis promotes Sirtuin-1 degradation, but soluble RAGE (sRAGE) acts as a decoy receptor, modulating this signaling pathway. In this hypothesis-generating study, we explore whether relatively higher versus lower body mass index (BMI) phenotypes are associated with differential sRAGE responses to caffeinated and decaffeinated coffee in patients with coronary artery disease (CAD). Methods: Thirty patients were allocated into two intervention-sequence groups that differed in mean BMI (*n* = 15). Group A (higher BMI) received caffeinated coffee followed by decaffeinated coffee; group B (lower BMI) received the inverse sequence. Biomarkers were measured at baseline, 28, and 56 days. Multiple regression models were constructed using sRAGE as the dependent variable and glucose, glycated hemoglobin (HbA1c), homocysteine, lipoprotein(a) (Lp(a)), Sirtuin-1, and small dense LDL (sdLDL) as predictors. Results: Serum biomarkers remained unchanged; only Sirtuin-1 increased after caffeinated coffee (*p* = 0.033) in group B. Regression analyses revealed distinct metabolic profiles between groups. Group A: Baseline sRAGE was inversely associated with glucose and Lp(a) (R^2^ = 0.748; *p* = 0.009). After coffee exposure, these associations disappeared. In group B, homocysteine and sRAGE were associated across all conditions. Furthermore, sRAGE was positively associated with sdLDL and inversely with HbA1c and Sirtuin-1 (R^2^ = 0.862; *p* = 0.021) after decaffeinated coffee. After caffeinated coffee, sRAGE was positively associated with glucose and SIRT-1, and inversely associated with Lp(a) (R^2^ = 0.908; *p* = 0.009). Conclusions: BMI appears to modulate the sRAGE response to coffee intake in CAD patients, with leaner individuals exhibiting a response that is highly dependent on the type of coffee consumed.

## 1. Introduction

Coffee, one of the most widely consumed beverages worldwide, valued for its unique taste and aroma, has played an important role in cultural traditions for centuries [1,2]. Coffee is a complex mixture whose best-known component is caffeine. On the other hand, coffee contains over 1000 chemical compounds, enabling multiple health benefits. Among all reported compounds, chlorogenic acid, caffeic acid, alkaloids, diterpenes such as cafestol and kahweol, lactones, vitamins, and minerals such as magnesium and potassium are the most studied [3,4].

The presence of several components in coffee suggests that habitual consumption is associated with many biological functions, including reduced risk of several chronic and degenerative diseases [5]. The beneficial effects of coffee have been studied in nonalcoholic fatty liver disease (NAFLD) [6], some cancers [7], Alzheimer’s disease [8], type 2 diabetes [9], and cardiovascular disease (CVD) [10]. Despite the benefits, heavy coffee consumption has been associated with an increased risk of arrhythmias [11] and higher blood pressure [12]. Molecular mechanisms underlying the cardiometabolic effects of coffee consumption are receiving attention, particularly pathways related to inflammation, oxidative stress, and advanced glycation.

Sirtuin-1 (Sirt-1), a NAD^+^-dependent deacetylase, plays a central role in glucose and lipid metabolism, insulin sensitivity, mitochondrial function, and inflammatory responses. Its activity is influenced by lifestyle factors, including diet, and can be stimulated by bioactive compounds such as polyphenols [13,14]. In a previous study from our group that evaluated the consumption of different types of coffee in healthy individuals, it was demonstrated that Sirt-1 is associated with coffee consumption [15], suggesting that coffee bioactive compounds may modulate early metabolic regulatory pathways. On the other hand, experimental evidence suggests that activation of the AGEs–RAGE (Advanced Glycation End-products–Receptor for Advanced Glycation End-products) axis may promote Sirt-1 degradation via ubiquitin–proteasome pathways, linking advanced glycation signaling to impaired metabolic regulation [16]. In this context, the interaction between AGEs and their receptor, RAGE, represents an important signaling axis involved in vascular inflammation and endothelial dysfunction [17]. RAGE, a member of the pattern-recognition receptor family, can exist in transmembrane and soluble forms [18].

Soluble RAGE (sRAGE) is detected in the circulation and body fluids. This presentation lacks intracellular domains and cannot initiate cell signaling. At least two isoforms of sRAGE have been described: cleaved (cRAGE) and endogenous secretory RAGE (esRAGE). The action of proteases at the cell surface, such as matrix metalloproteinases (MMPs) and disintegrin and metalloproteinase domain-containing protein 10 (ADAM10), results in the production of cRAGE. On the other hand, esRAGE is produced by alternative splicing of the RAGE gene [19].

The sRAGE isoform acts as a circulating decoy, modulating AGE–RAGE signaling. It is capable of inhibiting the binding of AGEs to their receptors in the cell membrane, thereby inhibiting the inflammatory response that usually follows their activation. Circulating levels of this biomarker are inversely associated with coronary heart disease in nondiabetic men [20] as well as with atherosclerosis [21] and left ventricular hypertrophy [22].

Elevated BMI is closely linked to chronic low-grade inflammation, insulin resistance, oxidative stress, and activation of the AGE–RAGE axis [23]. Circulating sRAGE has been proposed as a biomarker related to these metabolic phenotypes, and higher sRAGE concentrations have been reported in association with lower BMI [24,25]. In parallel, moderate coffee consumption is generally considered safe and has been associated with potentially favorable cardiometabolic effects [26]. Because metabolic phenotypes may modulate individual responses to dietary interventions, the magnitude and direction of the sRAGE response to coffee could differ according to BMI-related metabolic status. Therefore, this study aimed to explore whether relatively higher versus lower BMI phenotypes were associated with differential sRAGE responses to caffeinated and decaffeinated coffee in patients with coronary artery disease.

## 2. Material and Methods

### 2.1. Participants and Study Design

The present study was developed at the Instituto do Coração (InCor) do Hospital das Clinicas da Faculdade de Medicina da Universidade de São Paulo—HCFMUSP, São Paulo, SP, Brazil. Thirty individuals, aged from 40 to 80 years old with coronary artery disease (CAD) confirmed by cinecoronariography, were included to evaluate the effects of coffee consumption on metabolic profile. The exclusion criteria were: chronic renal failure (serum creatinine ≥ 2.0 mg/dL); fasting glucose ≥ 126 mg/dL; glycated hemoglobin ≥ 6.5%; heart electrical conduction disturbances; and ventricular dysfunction (≤40%). Other exclusion criteria were any previous self-reported history of alcohol dependence, smokers, liver failure, hematological disorders, or presence of metabolic disorders. This work was conducted as a specific sub-study focusing on advanced molecular biomarkers, derived from a larger primary clinical trial registered at ClinicalTrials.gov (Registry Number: NCT01003392, 7 January 2014).

This is a hypothesis-generating study in which patients were allocated to two intervention-sequence groups that differed in mean BMI. Group A had a higher mean BMI than group B. Rather than defining mutually exclusive clinical BMI categories, this division allowed us to explore whether sRAGE responses to coffee exposure differ between relatively higher and lower BMI phenotypes.

**Figure 1:**
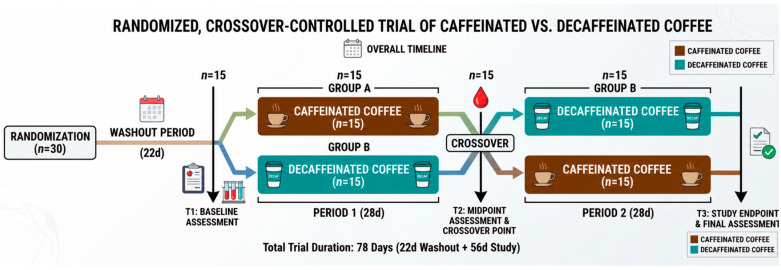
Schematic timeline of the randomized, crossover-controlled trial. The diagram details the study phases, sample sizes (total *n* = 30; *n* = 15 per group), exposure order, and assessment timing throughout the 78-day protocol. Following an initial 22-day washout, patients underwent baseline assessment (T1) and were allocated to either Sequence Group A (caffeinated followed by decaffeinated coffee) or Sequence Group B (decaffeinated followed by caffeinated coffee) for two consecutive 28-day periods. The midpoint assessment (T2) serves as the crossover point without an intermediate washout, acting as a critical checkpoint to evaluate potential carryover or cumulative effects before the intervention switch, which concludes at the final assessment (T3). Note: Infographic generated using AI tools based on author-defined prompts; no scientific interpretation was automated.

After a 22-day washout, patients were randomized to the sequence of coffee exposure in a crossover design: 28 days of caffeinated coffee (Nespresso^®^ (Lausanne, Switzerland) Volluto) followed by 28 days of decaffeinated coffee (Nespresso^®^ Volluto Decaffeinated), or the reverse sequence to evaluate if there is a difference in caffeinated or decaffeinated coffee, without a washout before crossover. Patients’ follow-up was performed at baseline (T1), after the first intervention period (T2), and after the second intervention period (T3). This approach allowed evaluation of within-individual coffee effects while treating BMI as a potential modifier of sRAGE-related metabolic outcomes (Figure 1).

During the study follow-up, consumption of foods such as cocoa, guarana, chocolate, and tea was prohibited. Participants were also instructed to maintain their routine dietary and physical activity habits. Also, they were instructed on the quantity and type of coffee to consume during specific phases of the study, and to return the used capsules to assess adherence. Compliance was continuously monitored by the research team during clinical visits.

Participants aged 60 or younger were instructed to drink 4 capsules of coffee per day (totaling around 400 mg of caffeine), while participants aged 60 or older were instructed to drink 3 capsules per day (totaling around 240 mg of caffeine) due to reduced tolerance to caffeine [27]. Blood samples were collected, weight was measured, and BMI was calculated at visit T1 (basal), after 28 days (T2), and after 56 days (T3) from the start of coffee intake. All subjects provided written informed consent. The research protocol was approved by the Ethics Committee of the University of São Paulo Medical School, Brazil (Approval Code: 92029118.1.0000.0068, on 20 July 2018), in accordance with the Declaration of Helsinki.

Venous blood was drawn after 12 h of fasting. Serum total cholesterol, triglycerides, High-Density Lipoprotein (HDL-c), and glucose were obtained by commercial colorimetric–enzymatic methods. Low-density lipoprotein (LDL-c) was calculated using the Friedwald equation. Measurements were performed using Dimension RxL equipment (Siemens Healthcare, Erlangen, Germany) with dedicated reagents. Apolipoproteins A and B, lipoprotein a (Lp(a), and glycated hemoglobin (HbA1c) were obtained by immunonephelometry using dedicated reagents for BN-II equipment from Siemens Healthcare. Plasma total homocysteine concentrations were measured by a chemiluminescence immunoassay on an Advia Centaur^®^ Analyzer (Siemens Healthcare Diagnostic). Commercial ELISA kits were used to measure serum concentrations of Sirt-1 (Novus Biologicals, Cambridge, UK; intra-assay CV = 5.7%, inter-assay CV = 5.2%), small dense LDL (sdLDL; R&D Systems, Minneapolis, MN, USA; intra-assay CV = 4.3%, inter-assay CV = 3.9%), and total sRAGE (R&D Systems; intra-assay CV = 6.2%, inter-assay CV = 8.3%). The analyses were performed in a blinded manner and according to the manufacturer’s instructions. Optical density was measured using a Multiscan FC plate reader (Thermo Fisher Scientific, Waltham, MA, USA).

### 2.2. Statistical Analyses

To determine the sample size, a two-tailed paired t-test was considered, with a significance level of 5% (alpha = 0.05) and 80% power. Based on previous data, in which the baseline level of Sirt-1 was 0.50 ± 0.10 ng/mL, a 20% increase (a difference of 0.10 ng/mL) was estimated following the intervention [15]. Assuming a moderate correlation (r = 0.5) between the pre- and post-intervention measurements, the calculated sample size was 10 patients. Accounting for a 20% safety margin to accommodate potential losses to follow-up, the final established sample size was 12 patients.

Participants were randomly assigned using SPSS (version 20). It was a double-blind protocol, as both participants and assessors were blinded to the group allocation. Kolmogorov–Smirnov’s distribution test was used to assess the distribution of the variables. Baseline and post-treatment variables were summarized using descriptive statistics and reported as means and standard deviations (SDs) or medians and interquartile ranges (IQRs) according to the distribution. A paired-sample *t*-test and Wilcoxon’s test were used for pre- and post-treatment comparisons. Independent t-tests and Mann–Whitney, ANOVA, and Friedman tests were used for comparisons between groups. Multiple least squares regression was performed using the backward elimination method, with sRAGE as the dependent variable and HbA1c, glucose, homocysteine, Sirt-1, sdLDL, and Lp(a) as independent variables. For multiple regression analysis, we calculated the coefficient of determination (R^2^), standardized regression coefficients (β), unstandardized regression coefficients (B), and standard errors (SEs). The variance inflation factor (VIF) was used to assess potential multicollinearity among independent variables, and values below 5 were considered indicative of adequate multicollinearity. Residual independence was assessed using the Durbin–Watson statistic, where values between 1.5 and 2.5 were considered indicative of acceptable residual independence. Residual normality was evaluated using the Shapiro–Wilk test, and a normal distribution was assumed for *p*-values > 0.05. Statistical significance was established at *p* < 0.05. Statistical analyses were performed using MedCalc Statistical Software, version 22.014.

## 3. Results

### 3.1. Population Characteristics and Anthropometric Measurements

The study population was predominantly male (96.7%), with a mean age of 62.9 ± 8.5 years. Nutritional status assessment revealed that 46.3% of participants were classified as overweight (BMI 25.0–29.9) and 20% as obese (BMI > 30.0). At the end of the study, there were no significant changes in weight, BMI, or blood pressure (Table 1).

Regarding the health history of the participants, the most prevalent clinical conditions were previous myocardial infarction (67%), overweight (63.3%), and hypertension (60.7%), as shown in Table 2. Regarding pharmacological treatment, all patients were receiving atorvastatin and acetylsalicylic acid (Table 2).

The standardized coffee intervention protocol was well tolerated by all participants, and no adverse events or clinical complications were reported during either study phase.

### 3.2. Biochemical Analysis

Group A had a higher mean BMI than group B (28.2 ± 4.6 kg/m^2^, range 20.9–37.4 kg/m^2^ vs. 25.2 ± 3.0 kg/m^2^, range 19.5–29.4 kg/m^2^, respectively, *p* = 0.03), although individual BMI values overlapped between groups. Thus, the comparison reflects intervention-sequence groups with relatively different mean BMI values, rather than two discrete BMI categories. Biochemical variables were determined for the two groups at baseline (T1). No statistically significant differences were observed between the two groups (Table 3).

Caffeinated and Decaffeinated Coffee Consumption: A Comparative Analysis of Groups A and B.

**Figure 2:**
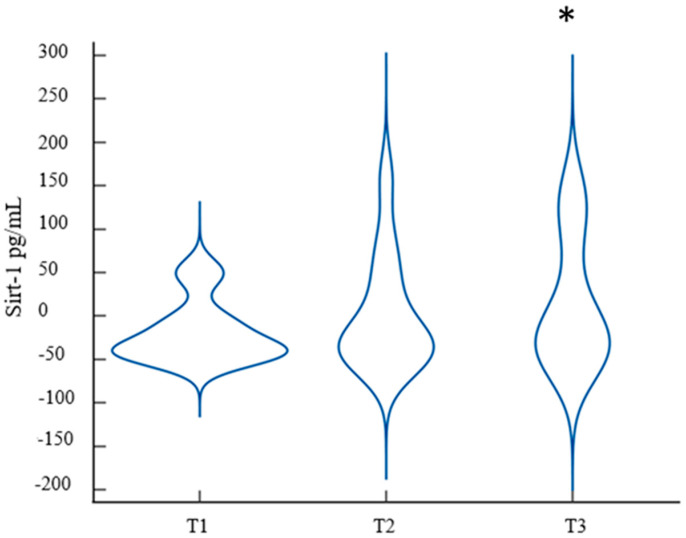
Violin plot of Sirt-1 concentrations of group B at the three time points. T1—baseline; T2—decaffeinated coffee; T3—caffeinated coffee. The plot was generated using the Friedman test and a significant difference was observed between T3 and T1 (* *p* = 0.03).

Biochemical variables were analyzed in groups A and B, and no significant differences were observed across the three assessment time points (T1, T2, and T3) for either group (Table 4 and Table 5). The sole exception was Sirt-1, which exhibited an increase in concentration following caffeinated coffee consumption (T3) in group B relative to baseline (Figure 2).

Multiple regression analysis was performed to assess the influence of HbA1c, glucose, homocysteine, Sirt-1, sdLDL, and Lp(a) on sRAGE levels in group A at T1, T2, and T3.

According to our results, baseline sRAGE was inversely associated with glucose (β = −1.06; B ± SE = −6.94 ± 1.78; *p* = 0.004; VIF = 2.840) and Lp(a) (β = −0.91; B ± SE = −2.13 ± 0.47; *p* = 0.002; VIF = 1.406), with a final R^2^ = 0.748 (*p* = 0.009), but with no significant associations after either coffee exposure.

The same multiple regression model was applied to group B. At baseline, sRAGE was positively associated with homocysteine (R^2^ = 0.722; *p* = 0.040). After 28 days of decaffeinated coffee intake, sRAGE was positively associated with homocysteine and sdLDL and inversely associated with glycated hemoglobin and Sirt-1 (R^2^ = 0.862; *p* = 0.021). After an additional 28 days of caffeinated coffee intake, sRAGE was positively associated with glucose, homocysteine, and Sirt-1 and inversely with Lp(a) (R^2^ = 0.908; *p* = 0.009). Multicollinearity diagnostics were assessed using variance inflation factors (VIFs), with values <5 indicating acceptable independence among predictors (Table 6).

Assumptions of the regression models were examined through residual diagnostics. Residuals showed a normal distribution according to the Shapiro–Wilk test (all *p* > 0.05), and independence of residuals was confirmed by the Durbin–Watson statistic (DW values ranging from 1.70 to 2.42) (provided in Supplementary Materials), supporting the adequacy of all fitted models.

## 4. Discussion

In the present exploratory crossover study, participants were assigned to two intervention-sequence groups that differed in mean BMI. Thus, BMI was considered a phenotypic characteristic that could influence the sRAGE-related response, rather than a strictly obesity-based stratification variable. Groups A and B received caffeinated and decaffeinated coffee in a crossover sequence. After 56 days of intervention, coffee consumption did not promote statistically significant changes in classical lipid metabolism biomarkers (total cholesterol, LDL-c, HDL-c, triglycerides, Lp(a), and small LDL) or glucose metabolism biomarkers (fasting glucose, HbA1c, and sRAGE). Despite no changes in traditional metabolic biomarkers, coffee consumption was associated with a significant increase in circulating Sirt-1 levels only in the lower BMI group, suggesting a potential early molecular effect of coffee on metabolic regulation. Also, sRAGE was associated with inflammatory markers, glucose, and lipid metabolism.

Sirt-1 is considered a master metabolic regulator and plays a key role in a wide range of pathologies and biological processes, such as obesity, diabetes, inflammation, cardiovascular disease, and aging [28,29]. Caloric restriction, physical activity, and natural compounds such as polyphenols can stimulate Sirt-1 levels [13,30]. Caffeine is the main bioactive compound in coffee, but it also contains hundreds of other bioactive phytochemicals, including polyphenols such as chlorogenic acid and lignans [31]. In the present work, Sirt-1 levels in group B were significantly increased after 56 days of coffee intake; however, group A did not show the same increase despite coffee consumption. These differences in responses to coffee intake may be attributed to group A patients having a higher BMI. Deleted in breast cancer–1 protein (DCB-1) can bind directly to the catalytic domain of Sirt-1, preventing substrate binding to Sirt-1 with inhibition of its activity. The weaker response in the higher BMI group might be due to obesity-related issues, such as metabolic stress driven by chronic inflammation (AGE–RAGE), which increases the interaction between Sirt-1 and DBC-1, thereby decreasing Sirt-1 deacetylase activity [32,33]. However, since we did not directly measure DBC1 or tissue-specific RAGE, this theory is just a starting point and needs to be tested in larger molecular studies.

Beyond this possible impairment in Sirt-1 response, obesity indices were also associated with sRAGE levels. sRAGE, which can be found in plasma and other body fluids, has the same ligand-binding specificity as RAGE and may act as a ‘decoy’ by binding proinflammatory ligands and preventing them from reaching membrane RAGE, mitigating the deleterious effects of the activation of the full-length receptor [34]. Studies have shown that reduced levels of sRAGE are independently associated with CAD and inversely correlated with BMI and waist-to-hip ratio in metabolic syndrome [20,25].

Our results suggest that in higher BMI individuals, sRAGE may not respond to bioactive compounds after coffee intake. Multiple regression analyses showed that in group A, baseline sRAGE was inversely associated with glucose and Lp(a), but after coffee exposure, these associations no longer persisted. This initial inverse association suggests a compensatory decoy behavior, where sRAGE acts as a passive counter-regulatory marker of chronic cardiometabolic burden. However, after coffee exposure and the inclusion of polyphenols and caffeine in the system, the previous associations were lost. This likely reflects a higher BMI phenotype, in which coordinated communication among key metabolic pathways becomes dysregulated. Indeed, obesity is now recognized as a complex network disease involving neuroendocrine, adipose, immune, and gut microbiome dysregulation with impaired organ crosstalk. This pathology is driven by chronic low-grade inflammation sustained through immunometabolic reprogramming, accelerating biological aging via cellular senescence, mitochondrial dysfunction, oxidative stress, and a loss of metabolic resilience [35].

In group B, the results of multiple regression analyses showed considerable differences between the two groups. In group B, homocysteine was an independent variable systematically associated with sRAGE. The positive association was present at baseline, after decaffeinated coffee, and after caffeinated coffee consumption. Homocysteine, an amino acid derived from methionine metabolism, has been considered an independent biomarker for cardiovascular disease due to its capacity to impair endothelial function, enhance oxidative stress and inflammation, stimulate vascular smooth muscle proliferation, and increase thrombogenicity [36]. Hyperhomocysteinemia was associated with increased expression of RAGE and its key ligands [37]. From a clinical perspective, this stable association between homocysteine and sRAGE in the lower BMI population has important risk-assessment value. In non-obese CAD patients, traditional adipose-centric inflammatory markers are often absent. In this specific phenotype, elevated homocysteine may drive baseline endothelial oxidative stress, triggering a continuous, compensatory shedding of membrane RAGE into soluble sRAGE to act as a protective decoy, once stimulated by inflammatory signals [38]. Therefore, evaluating the homocysteine–sRAGE axis could serve as a valuable clinical tool to identify residual cardiovascular risk and track subclinical vascular deterioration in CAD patients who appear metabolically low-risk due to lower BMI.

Also, in group B, sRAGE was positively associated with sdLDL and inversely associated with HbA1c and Sirt-1 after 28 days of decaffeinated coffee. When the crossover occurred, and the patients consumed an additional 28 days of caffeinated coffee, the results changed. The association with Sirt-1 became positive, adding to the positive association with glucose and the negative association with Lp(a). These results suggest that decaffeinated and caffeinated coffee may exert partially distinct metabolic effects.

Both caffeinated and decaffeinated coffee were associated with reduced diabetes risk [9]; however, decaffeinated coffee was associated with a more pronounced reduction in HbA1c in healthy individuals and those with diabetes mellitus [39]. The inverse association between sRAGE and HbA1c observed following decaffeinated coffee intake may indicate improved long-term glycemic control, apparently dissociated from Sirt-1-related adaptive pathways. Furthermore, the positive association detected between sRAGE and sdLDL may reflect the link between RAGE activation and lipoprotein burden.

In contrast, after crossover and introduction of caffeinated coffee, a reorganization of these associations was observed. Sirt-1 shifted from an inverse to a positive relationship with sRAGE, a positive association with glucose, and an inverse association with Lp(a). These changes in the associations between biochemical markers after caffeinated coffee could be justified by the introduction of caffeine into the system. Caffeine has been shown to activate energy-sensing pathways, enhancing AMPKα phosphorylation levels and Sirt1 expression [40]. RAGE-Sirt-1 coupling observed after caffeinated coffee suggests the activation of an adaptive RAGE–Sirt-1 response. The absence of a formal washout period prior to the crossover could suggest that the observed effect of caffeinated coffee may partially reflect the cumulative or carryover effects of continuous coffee consumption. However, the intermediate measurement (T2) before switching coffee types allowed for the evaluation of biomarker evolution and the capture of the dynamic transition between interventions.

Metabolomic data indicate that coffee consumption is associated with reduced levels of diacylglycerols and triacylglycerols—key mediators of lipotoxicity and insulin resistance [41]. Lp(a) is an LDL (low-density lipoprotein) cholesterol variant, mostly determined on genetic bases, that can affect the estimated risk of cardiovascular disease through several mechanisms involving proinflammatory and prothrombotic pathways [42]. Oxidized phospholipids present in Lp(a) may act as damage-associated molecular patterns (DAMPs), molecules that bind to membrane-bound RAGE and activate inflammation [43]. The inverse association between these two variables seems consistent with differences in timing and biological compartmentalization, with Lp(a) representing a stable, chronic lipoprotein-driven vascular injury and RAGE representing the dynamic regulatory response of the AGE-RAGE axis.

Together, these results suggest that caffeine may improve metabolic parameters of lipid and glycemic pathways.

## 5. Limitations of the Study

One limitation of the study is the lack of a washout period between the two coffee interventions. Thus, the observed responses may reflect the interaction between 56 days of cumulative coffee exposure and the underlying metabolic phenotype, rather than a simple beverage-type effect. The relatively small sample size may have limited the statistical power to identify differences in biochemical markers. Additionally, this limited sample size may have affected the overall stability of our multiple regression models and increased the risk of overfitting. While our rigorous diagnostic criteria (VIF, Durbin–Watson, and Shapiro–Wilk) confirmed the mathematical validity of these models within our cohort, these findings should be treated as exploratory. Furthermore, the follow-up period may not have been sufficient to induce significant changes in serum biomarkers, such as those related to lipid and glucose metabolism, which often require longer-term exposure to reflect consistent changes. Another point that must be taken into consideration is that the participants are under pharmacological treatment with statins and hypoglycemic drugs, and these medications could have disturbed the effects of coffee consumption. Additionally, a controlled AGE-restricted diet was not implemented, and a quantitative assessment of AGE intake within participants’ habitual dietary patterns was not performed. Finally, circulating sRAGE was used as a systemic biomarker of RAGE pathway activity. Plasma measurements do not capture tissue-specific receptor expression, isoform distribution (cRAGE vs. esRAGE), or metalloproteinase-mediated shedding dynamics, which may differ across metabolic phenotypes. Mechanistic studies integrating cellular and molecular endpoints are warranted to further clarify these pathways.

## 6. Conclusions

In conclusion, despite the small sample size in this study, our exploratory findings suggest that the sRAGE-Sirtuin-1 axis may exhibit distinct association patterns across BMI phenotypes following coffee consumption. Specifically, homocysteine emerges as an upstream determinant of RAGE signaling only in leaner individuals after consuming either caffeinated or decaffeinated coffee. Since this is a hypothesis-generating study, these results require further validation through cellular experimentation, animal models, and subsequent large-sample cohort studies.

## Figures and Tables

**Table 1:** Clinical characteristics of the study population.

Clinical Characteristics	Baseline (*n* = 30)	End of Study (*n* = 30)
Weight (kg)	75.37 ± 12.27	74.2 ± 12.31
Body Mass Index	26.7 ± 3.98	26.3 ± 4.22
Systolic blood pressure (mmHg)	127 ± 16.7	123 ± 13.2
Diastolic blood pressure (mmHg)	77 ± 9.1	75 ± 9.3

Data are mean (SD).

**Table 2:** Clinical characteristics and medications in use by patients.

Comorbidities	(%)
Hypertension	60.7
Diabetes mellitus (history without medication)	21.4
Overweight or Obesity	63.3
Myocardial infarction	67.9
Angina pectoris	32.1
**Medication**	
Atorvastatin	100.0
Acetylsalicylic acid	100.0
Angiotensin-Converting Enzyme inhibitors	35.7
Beta blockers	67.9
Angiotensin II receptor blockers	28.6
Amlodipine	32.1

**Table 3:** Baseline characteristics of the participants, comparison between groups A and B.

Variables (*n* = 15)	Group A T1 (Basal)	Group B T1 (Basal)
Age (year)	61 ± 8.3	65 ± 8.5
Sex (male/female)	15/0	14/1
Total cholesterol (mg/dL)	141 ± 34.7	136 ± 28.8
HDL (mg/dL)	46 ± 15.6	44 ± 6.6
LDL (mg/dL)	77 ± 26.0	72 ± 20.8
Triglycerides (mg/dL)	92 ± 36.6	95 ± 42.2
Apolipoprotein A1 (mg/mL)	1.29 ± 0.28	1.31 ± 0.20
Apolipoprotein B (mg/mL)	0.69 ± 0.19	0.70 ± 0.18
Lipoprotein (a) (mg/dL)	60.0 (14.8–93.5)	22 (8.0–33.0)
Small dense LDL (nmol/mL)	3.7 (2.1–4.5)	5.7 (2.4–8.8)
Homocysteine (µmol/L)	12.0 (10.0–14.8)	12.5 (11.0–15.0)
Glucose (mg/dL)	104 ± 14.4	97 ± 11.3
Glycated hemoglobin (%)	5.5 ± 0.55	5.6 ± 0.39
sRAGE (ng/mL)	262 ± 94.6	303 ± 109.8
Sirtuin-1 (pg/mL)	23.0 (14.0–40.0)	14.5 (7.0–44.5)

Data are presented as mean (SD) or median (IQR); sRAGE: soluble receptor for advanced glycation end-products; *p*-values were obtained using independent *t*-test and Mann–Whitney test.

**Table 4:** Comparison among biochemical markers in group A.

Variable (*n* = 15)	T1 (Basal)	T2 (Caffeinated)	T3 (Decaffeinated)
Total cholesterol (mg/dL)	141 ± 34.7	143 ± 29.5	135 ± 33.9
HDL (mg/dL)	46 ± 15.6	46 ± 15.4	43 ± 14.1
LDL (mg/dL)	77 ± 26.0	77 ± 24.8	74 ± 24.9
Triglycerides (mg/dL)	92 ± 36.6	97 ± 49.7	92 ± 42.4
Apolipoprotein A1(mg/mL)	1.29 ± 0.28	1.37 ± 0.21	1.33 ± 0.34
Apolipoprotein B (mg/mL)	0.69 ± 0.19	0.71 ± 0.19	0.67 ± 0.19
Lipoprotein (a) (mg/dL)	60.0 (14.8–93.5)	63.0 (11.8–82.3)	56.8 (12.2–83.3)
Small dense LDL (nmol/mL)	3.7 (2.1–4.5)	3.7 (3.3–4.9)	4.4 (2.3–9.5)
Homocysteine (µmol/L)	12.0 ± 3.4	11.6 ± 2.5	11.2 ± 2.9
Glucose (mg/dL)	104 ± 14.4	105 ± 14.7	106 ± 14.9
Glycated hemoglobin (%)	5.5 ± 0.55	5.6 ± 0.55	5.6 ± 0.45
sRAGE (ng/mL)	262 ± 94.6	245 ± 73.3	269 ± 88.6
Sirtuin-1 (pg/mL)	23.0 (14.5–40.0)	23.5 (10.0–62.0)	30.0 (25.7–54.5)

Data are presented as mean and standard deviation (±SD); sLDL and Sirt-1 data are median and interquartile rage (IQR).

**Table 5:** Comparison among biochemical markers in group B.

Variable (*n* = 15)	T1 (Basal)	T2 (Decaffeinated)	T3 (Caffeinated)	*p* *
Total cholesterol (mg/dL)	136 ± 28.8	143 ± 34.3	144 ± 31.2	0.371
HDL (mg/dL)	44 ± 6.6	46 ± 7.9	46 ± 9.2	0.200
LDL (mg/dL)	72 ± 20.8	79 ± 25.9	75 ± 23.3	0.593
Triglycerides (mg/dL)	95 ± 42.2	99 ± 41.1	112 ± 45.3	0.234
Apolipoprotein A1 (mg/mL)	1.31 ± 0.20	1.37 ± 0.20	1.38 ± 0.23	0.054
Apolipoprotein B (mg/mL)	0.70 ± 0.18	0.70 ± 0.14	0.72 ± 0.16	0.771
Lipoprotein (a) (mg/dL)	22.0 (8.0–33.0)	23.0 (16.0–69.0)	15.0 (10.5–60.6)	0.983
Small dense LDL (nmol/mL)	5.7 (2.4–8.8)	2.2 (1.5–9.7)	4.2 (2.7–9.9)	0.578
Homocysteine (µmol/L)	12.5 (11.0–15.0)	13.0 (10.0–15.0)	10.8 (10.4–14.3)	0.749
Glucose (mg/dL)	97 ± 11.3	100 ± 8.8	99 ± 9.2	0.377
Glycated hemoglobin (%)	5.6 ± 0.39	5.6 ± 0.35	5.5 ± 0.38	0.059
sRAGE (ng/mL)	303 ± 109.8	305 ± 127.3	325 ± 125.6	0.072
Sirtuin-1 (pg/mL)	14.5 (7.0–44.5)	17.0 (10.3–108.5)	28.0 (10.0–126.0)	0.033 *

Data are mean presented as mean and standard deviation (SD); small dense LDL and Sirtuin-1 data are median and interquartile rage (IQR); * *p*-value was obtained from ANOVA and Friedman tests.

**Table 6:** Multiple regression analysis in group B.

Variable	β	B	SE	*p*	VIF
Baseline					
Homocysteine	0.680	10.948	3.384	0.014	1.203
Decaffeinated Coffee					
HbA1c	−1.12	−403.8	84.9	0.003	2.368
Homocysteine	0.93	16.8	3.562	0.003	1.803
Sirt-1	−1.02	−0.759	0.192	0.008	3.101
sdLDL	1.22	31.73	7.25	0.005	3.394
Caffeinated Coffee					
Glucose	0.546	7.453	2.227	0.016	1.886
Homocysteine	0.911	12.526	1.924	0.001	1.394
Lp(a)	−0.401	−1.486	0.533	0.032	1.417
Sirt-1	0.736	1.268	0.287	0.005	1.801

β: standardized regression coefficient; B: unstandardized regression coefficient; SE: standard error; and VIF: variance inflation factor.

## Data Availability

The raw data supporting the conclusions of this article will be made available by the authors on request due to privacy concerns, as they contain sensitive personal information that could compromise participant confidentiality.

## Supplementary Materials

The following supporting information can be downloaded at: https://www.mdpi.com/article/10.3390/nu18142305/s1, Table S1: Multiple least squares regression. Table S2: Assessment of normality of residuals from multiple regression models using the Shapiro–Wilk test. Table S3: Assessment of residual independence in multiple regression models using the Durbin–Watson statistic.

## References

[1] Nieber K. (2017). The impact of coffee on health. Planta Med..

[2] Chieng D., Kistler P.M. (2022). Coffee and tea on cardiovascular disease (CVD) prevention. Trends Cardiovasc. Med..

[3] Baspinar B., Eskici G., Ozcelik A.O. (2017). How coffee affects metabolic syndrome and its components. Food Funct..

[4] Machado F., Coimbra M.A., Del Castillo M.D., Coreta-Gomes F. (2023). Mechanisms of action of coffee bioactive compounds—A key to unveil the coffee paradox. Crit. Rev. Food Sci. Nutr..

[5] Ding Q., Xu Y.M., Lau A.T.Y. (2023). The epigenetic effects of coffee. Molecules.

[6] Kositamongkol C., Kanchanasurakit S., Auttamalang C., Inchai N., Kabkaew T., Kitpark S., Chaiyakunapruk N., Duangjai A., Saokaew S., Phisalprapa P. (2021). Coffee consumption and non-alcoholic fatty liver disease: An umbrella review and a systematic review and meta-analysis. Front. Pharmacol..

[7] Pauwels E.K.J., Volterrani D. (2021). Coffee consumption and cancer risk: An assessment of the health implications based on recent knowledge. Med. Princ. Pract..

[8] Yelanchezian Y.M.M., Waldvogel H.J., Faull R.L.M., Kwakowsky A. (2022). Neuroprotective effect of caffeine in Alzheimer’s disease. Molecules.

[9] Ding M., Bhupathiraju S.N., Chen M., van Dam R.M., Hu F.B. (2014). Caffeinated and decaffeinated coffee consumption and risk of type 2 diabetes: A systematic review and dose-response meta-analysis. Diabetes Care.

[10] Ding M., Bhupathiraju S.N., Satija A., van Dam R.M., Hu F.B. (2014). Long-term coffee consumption and risk of cardiovascular disease: A systematic review and dose-response meta-analysis of prospective cohort studies. Circulation.

[11] Surma S., Romańczyk M., Filipiak K.J., Lip G.Y.H. (2023). Coffee and cardiac arrhythmias: Update review of the literature and clinical studies. Cardiol. J..

[12] Jee S.H., He J., Whelton P.K., Suh I., Klag M.J. (1999). The effect of chronic coffee drinking on blood pressure: A meta-analysis of controlled clinical trials. Hypertension.

[13] Mansur A.P., Roggerio A., Goes M.F.S., Avakian S.D., Takada J.Y., Strunz C.M.C. (2017). Gene expression of sirtuin-1 and receptor for advanced glycation end products in healthy and slightly overweight subjects after caloric restriction and resveratrol administration. Eur. Heart J..

[14] Grabowska W., Sikora E., Bielak-Zmijewska A. (2017). Sirtuins, a promising target in slowing down the ageing process. Biogerontology.

[15] Gonçalinho G.H.F., Nascimento J.R.O., Mioto B.M., Amato R.V., Moretti M.A., Strunz C.M.C., Cesar L.A.M., Mansur A.P. (2022). Effects of coffee on Sirtuin-1, homocysteine, and cholesterol in healthy adults: Does the coffee powder matter?. J. Clin. Med..

[16] Huang K.P., Chen C., Hao J., Huang J.Y., Liu P.Q., Huang H.Q. (2015). AGEs–RAGE system down-regulates SIRT1 through the ubiquitin–proteasome pathway to promote FN and TGF-β1 expression in male rat glomerular mesangial cells. Endocrinology.

[17] Ramasamy R., Yan S.F., Schmidt A.M. (2005). The RAGE axis and endothelial dysfunction: Maladaptive roles in the diabetic vasculature and beyond. Trends Cardiovasc. Med..

[18] Liliensiek B., Weigand M.A., Bierhaus A., Nicklas W., Kasper M., Hofer S., Plachky J., Gröne H.J., Kurschus F.C., Schmidt A.M. (2004). Receptor for advanced glycation end products (RAGE) regulates sepsis but not the adaptive immune response. J. Clin. Investig..

[19] Pinto R.S., Minanni C.A., de Araújo-Lira A.L., Passarelli M. (2022). Advanced glycation end products: A sweet flavor that embitters cardiovascular disease. Int. J. Mol. Sci..

[20] Falcone C., Emanuele E., D’Angelo A., Buzzi M.P., Belvito C., Cuccia M., Geroldi D. (2005). Plasma levels of soluble receptor for advanced glycation end products and coronary artery disease in nondiabetic men. Arterioscler. Thromb. Vasc. Biol..

[21] Basta G., Leonardis D., Mallamaci F., Cutrupi S., Pizzini P., Gaetano L., Tripepi R., Tripepi G., De Caterina R., Zoccali C. (2010). Circulating soluble receptor of advanced glycation end product inversely correlates with atherosclerosis in patients with chronic kidney disease. Kidney Int..

[22] Leonardis D., Basta G., Mallamaci F., Cutrupi S., Pizzini P., Gaetano L., Tripepi R., Tripepi G., De Caterina R., Zoccali C. (2012). Circulating soluble receptor for advanced glycation end product and left ventricular hypertrophy in patients with chronic kidney disease. Nutr. Metab. Cardiovasc. Dis..

[23] Vianello E., Beltrami A.P., Aleksova A., Janjusevic M., Fluca A.L., Corsi Romanelli M.M., La Sala L., Dozio E. (2025). The Advanced Glycation End-Products (AGE)-Receptor for AGE System (RAGE): An Inflammatory Pathway Linking Obesity and Cardiovascular Diseases. Int. J. Mol. Sci..

[24] Miranda E.R., Somal V.S., Mey J.T., Blackburn B.K., Wang E., Farabi S., Karstoft K., Fealy C.E., Kashyap S., Kirwan J.P. (2017). Circulating soluble RAGE isoforms are attenuated in obese, impaired-glucose-tolerant individuals and are associated with the development of type 2 diabetes. Am. J. Physiol. Endocrinol. Metab..

[25] Tayyib N.A., Ramaiah P., Alshahrani S.H., Margiana R., Almalki S.G., Kareem A.K., Zabibah R.S., Shbeer A.M., Ali S.H.J., Mustafa Y.F. (2023). Soluble receptor for advanced glycation end products (sRAGE) is associated with obesity rates: A systematic review and meta-analysis of cross-sectional study. BMC Endocr. Disord..

[26] Ungvari Z., Kunutsor S.K. (2024). Coffee consumption and cardiometabolic health: A comprehensive review of the evidence. GeroScience.

[27] Massey L.K. (1998). Caffeine and the elderly. Drugs Aging.

[28] Schug T.T., Li X. (2011). Sirtuin 1 in lipid metabolism and obesity. Ann. Med..

[29] Yang Y., Liu Y., Wang Y., Chao Y., Zhang J., Jia Y., Tie J., Hu D. (2022). Regulation of SIRT1 and its roles in inflammation. Front. Immunol..

[30] Zullo A., Simone E., Grimaldi M., Musto V., Mancini F.P. (2018). Sirtuins as mediators of the anti-ageing effects of calorie restriction in skeletal and cardiac muscle. Int. J. Mol. Sci..

[31] van Dam R.M., Hu F.B., Willett W.C. (2020). Coffee, caffeine, and health. N. Engl. J. Med..

[32] Kim J.E., Chen J., Lou Z. (2008). DBC1 is a negative regulator of SIRT1. Nature.

[33] Escande C., Chini C.C., Nin V., Dykhouse K.M., Novak C.M., Levine J., van Deursen J., Gores G.J., Chen J., Lou Z. (2010). Deleted in breast cancer-1 regulates SIRT1 activity and contributes to high-fat diet-induced liver steatosis in mice. J. Clin. Investig..

[34] Maillard-Lefebvre H., Boulanger E., Daroux M., Gaxatte C., Hudson B.I. (2009). Soluble receptor for advanced glycation end products: A new biomarker in diagnosis and prognosis of chronic inflammatory diseases. Rheumatology.

[35] Dutta G., Mishra P., Mishra S.P., Badhai J. (2026). Obesity as a Whole-Body Regulatory Disorder: A Systems Biology Framework for Metaflammation, Accelerated Aging, and Colorectal Cancer Risk. Onco.

[36] Weiss N., Keller C., Hoffmann U., Loscalzo J. (2002). Endothelial dysfunction and atherothrombosis in mild hyperhomocysteinemia. Vasc. Med..

[37] Hofmann M.A., Lalla E., Lu Y., Gleason M.R., Wolf B.M., Tanji N., Ferran L.J., Kohl B., Rao V., Kisiel W. (2001). Hyperhomocysteinemia enhances vascular inflammation and accelerates atherosclerosis in a murine model. J. Clin. Investig..

[38] Erusalimsky J.D. (2021). The use of the soluble receptor for advanced glycation-end products (sRAGE) as a potential biomarker of disease risk and adverse outcomes. Redox Biol..

[39] Archana D., Karthickeyan K. (2025). Impact of caffeine and decaffeinated coffee on blood glucose levels in healthy individuals and type 2 diabetes patients on antidiabetic medication. J. Neonatal Surg..

[40] Wang Y., Peng W., Li C., Yang J. (2025). Caffeine promotes adipocyte autophagy through the AMPK/SIRT1 signaling pathway and improves high-fat diet-induced obesity and leptin resistance. J. Food Biochem..

[41] Hang D., Zeleznik O.A., He X., Guasch-Ferré M., Jiang X., Li J., Liang L., Eliassen A.H., Clish C.B., Chan A.T. (2020). Metabolomic signatures of long-term coffee consumption and risk of type 2 diabetes in women. Diabetes Care.

[42] Greco A., Finocchiaro S., Spagnolo M., Faro D.C., Mauro M.S., Raffo C., Sangiorgio G., Capodanno D. (2025). Lipoprotein(a) as a pharmacological target: Premises, promises, and prospects. Circulation.

[43] Lin H., Xiong W., Fu L., Yi J., Yang J. (2025). Damage-associated molecular patterns (DAMPs) in diseases: Implications for therapy. Mol. Biomed..

