# Supplementary material for: BMI-Dependent Modulation of the Soluble RAGE-Sirtuin-1 Axis by Coffee Type in Coronary Artery Disease
Source: Nutrients. 2026 Jul 14;18(14):2305. doi: 10.3390/nu18142305 (PMC13415449; doi:10.3390/nu18142305)
Supplement: Supplementary file 1 [file nutrients-18-02305-s001.zip › nutrients-4387235-supplementary.pdf]

Supplementary Materials

Table S1. Multiple least squares regression for group A and Group B.

Group A

Condition	Predictor	B	SE	β	t	*p*	Partial r	Semi-partial r	VIF
R^2=0.748; adjusted R^2=0.636; F-ratio =6.67; P=0.0089									
Baseline	Glucose	-6.9414	1.7837	-1.059	-3.8915	0.0037	-0.792	0.6516	2.84
Baseline	HbA1c	105.2848	48.0609	0.609	2.1907	0.0562	0.5897	0.3668	2.548
Baseline	Lp(a)	-2.1324	0.472	-0.908	-4.5176	0.0015	-0.833	0.7564	1.406
Baseline	SIRT-1	0.3948	0.1907	0.383	2.0705	0.0683	0.568	0.3467	1.218

Group B

Condition	Predictor	B	SE	β	t	*p*	Partial r	Semi-partial r	VIF
Baseline: R^2=0.722; adjusted R^2=0.564; F-ratio=4.55; P=0.0399									
Baseline	HbA1c	-123.7	58.00	-0.46	-2.13	0.0704	-0.628	-0.425	1.178
Baseline	Homocysteine	10.95	3.38	0.68	3.24	0.0143	0.774	0.645	1.203
Baseline	SIRT-1	2.24	0.96	0.74	2.32	0.053	0.66	0.463	2.419
Baseline	sdLDL	-12.94	6.08	-0.68	-2.13	0.071	-0.626	-0.424	2.451

**Decaffeinated coffee: R^2=0.862; adjusted R^2=0.724; F-ratio=6.25; *p*=0.021**									
Decaffeinated coffee	Glucose	4.18	2.81	0.29	1.49	0.1873	0.519	0.226	1.769
Decaffeinated coffee	HbA1c	-403.8	84.88	-1.12	-4.76	0.0031	-0.889	-0.721	2.368
Decaffeinated coffee	Homocysteine	16.83	3.56	0.93	4.72	0.0032	0.888	0.716	1.803
Decaffeinated coffee	Lp(a)	-0.95	0.63	-0.27	-1.52	0.1804	-0.526	-0.23	1.411
Decaffeinated coffee	SIRT-1	-0.76	0.19	-1.02	-3.96	0.0075	-0.85	-0.6	3.101
Decaffeinated coffee	sdLDL	31.73	7.25	1.22	4.38	0.0047	0.873	0.664	3.394
**Caffeinated coffee: R^2=0.907; adjusted R^2=0.831; F-ratio=11.78; P=0.005**									
Caffeinated coffee	Glucose	7.453	2.23	0.55	3.35	0.0155	0.807	0.4155	1.886
Caffeinated coffee	HbA1c	113.2	46.54	-0.35	-2.43	0.0509	-0.7048	0.3021	1.431
Caffeinated coffee	Homocysteine	12.526	1.92	0.91	6.51	0.0006	0.936	0.8083	1.394
Caffeinated coffee	Lp(a)	-1.486	0.53	-0.4	-2.79	0.0317	-0.7512	0.346	1.417
Caffeinated coffee	SIRT-1	1.268	0.29	0.74	4.42	0.0045	0.8746	0.5484	1.801

Table S2. Assessment of normality of residuals from multiple regression models using the Shapiro–Wilk test.

Group A		
**Residuals**	**Shapiro-Wilk**	***p***
Baseline	W=0.9383	0.3967

Group B		
**Residuals**	**Shapiro-Wilk**	***p***
Baseline	W=0.9320	0.4022
Decaffeinated coffee	W=0.9423	0.4875
Caffeinated coffee	W=0.9721	0.9319

Table S3. Assessment of residual independence in multiple regression models using the Durbin–Watson statistic.

**Group A**	**Condition**	**Durbin–Watson test**
	Baseline (T1)	1.98
**Group B**	**Condition**	**Durbin–Watson test**
	Baseline (T1)	1.51
	Decaffeinated coffee (T2)	2.42
	Caffeinated coffee (T3)	1.70

Values close to 2.0 indicate independence of residuals. Values between 1.5 and 2.5 are generally considered acceptable, suggesting absence of relevant serial autocorrelation in regression residuals
